# Continuous versus bolus intermittent loop diuretic infusion in acutely decompensated heart failure: a prospective randomized trial

**DOI:** 10.1186/cc13952

**Published:** 2014-06-28

**Authors:** Alberto Palazzuoli, Marco Pellegrini, Gaetano Ruocco, Giuseppe Martini, Beatrice Franci, Maria Stella Campagna, Marilyn Gilleman, Ranuccio Nuti, Peter A McCullough, Claudio Ronco

**Affiliations:** 1 Department of Internal Medicine Cardiology Section, S Maria alle Scotte Hospital University of Siena, Siena, Italy; 2 Providence Hospitals and Medical Centers, Southfield, Novi, MI, USA; 3 Nephrology Dialysis & Transplantation International Renal Research Institute (IRRIV) St. Bortolo Hospital, Vicenza, Italy

## Abstract

**Introduction:**

Intravenous loop diuretics are a cornerstone of therapy in acutely decompensated heart failure (ADHF). We sought to determine if there are any differences in clinical outcomes between intravenous bolus and continuous infusion of loop diuretics.

**Methods:**

Subjects with ADHF within 12 hours of hospital admission were randomly assigned to continuous infusion or twice daily bolus therapy with furosemide. There were three co-primary endpoints assessed from admission to discharge: the mean paired changes in serum creatinine, estimated glomerular filtration rate (eGFR), and reduction in B-type natriuretic peptide (BNP). Secondary endpoints included the rate of acute kidney injury (AKI), change in body weight and six months follow-up evaluation after discharge.

**Results:**

A total of 43 received a continuous infusion and 39 were assigned to bolus treatment. At discharge, the mean change in serum creatinine was higher (+0.8 ± 0.4 versus -0.8 ± 0.3 mg/dl *P* <0.01), and eGFR was lower (-9 ± 7 versus +5 ± 6 ml/min/1.73 m^2^*P* <0.05) in the continuous arm. There was no significant difference in the degree of weight loss (-4.1 ± 1.9 versus -3.5 ± 2.4 kg *P* = 0.23). The continuous infusion arm had a greater reduction in BNP over the hospital course, (-576 ± 655 versus -181 ± 527 pg/ml *P* = 0.02). The rates of AKI were comparable (22% and 15% *P* = 0.3) between the two groups. There was more frequent use of hypertonic saline solutions for hyponatremia (33% versus 18% *P* <0.01), intravenous dopamine infusions (35% versus 23% *P* = 0.02), and the hospital length of stay was longer in the continuous infusion group (14. 3 ± 5 versus 11.5 ± 4 days, *P* <0.03). At 6 months there were higher rates of re-admission or death in the continuous infusion group, 58% versus 23%, (*P* = 0.001) and this mode of treatment independently associated with this outcome after adjusting for baseline and intermediate variables (adjusted hazard ratio = 2.57, 95% confidence interval, 1.01 to 6.58 *P* = 0.04).

**Conclusions:**

In the setting of ADHF, continuous infusion of loop diuretics resulted in greater reductions in BNP from admission to discharge. However, this appeared to occur at the consequence of worsened renal filtration function, use of additional treatment, and higher rates of rehospitalization or death at six months.

**Trial registration:**

ClinicalTrials.gov NCT01441245. Registered 23 September 2011.

## Introduction

The use of intravenous loop diuretics is a cornerstone of therapy for acutely decompensated heart failure (ADHF) treatment, especially in patients admitted with pulmonary congestion and volume overload. Significant concerns have been raised regarding the risks and benefits of loop diuretics, especially about the dosage and administration regimen [1,2]. Recent guidelines recommend the use of loop diuretics to reduce left ventricular filling pressure, avoid pulmonary edema, and alleviate peripheral fluid retention [3]. Therapeutic recommendations focus primarily on symptom relief because there are no specific strategies showing a clear benefit in the outcome of ADHF. Despite the ubiquity of loop diuretic administration, high quality data supporting their efficacy and best modality of administration are lacking. In addition, impaired renal function has consistently been proven to be an independent risk factor for adverse outcomes and a leading reason why higher doses and continuous infusions are used. The use of high doses of loop diuretics has been associated with unfavorable neuroendocrine activation, worsening renal function, electrolyte disturbances, and a poor outcome [4,5]. Although loop diuretics are the most commonly used drugs in ADHF treatment, their short- and long-term effects are relatively unknown; thus, it is recommended to administer the lowest dosage to patients with ADHF in order to relieve their symptoms [5]. Some studies have provided guidelines for the administration of these drugs in clinical practice, but data interpretation remains challenging due to the frequent exclusion of patients with kidney disease from major ADHF clinical trials. Therefore, it is not clear if continuous infusion is better than intermittent boluses in terms of decongestion, maintenance of renal filtration function, and prognosis [6,7]. In theory, intermittent boluses could lead to more unfavorable hemodynamic changes, be associated with a higher rate of diuretic resistance due to suboptimal drug levels in the renal tubules, and result in a rebound in sodium reabsorption. On the other hand, continuous administration should provide more constant delivery of diuretic into the tubule, potentially reducing this phenomenon. Additionally, continuous infusion may induce an increased dieresis, reducing systemic and pulmonary congestion more efficaciously, thus avoiding sudden blood pressure reduction induced by bolus administration. However, for the same reasons, continuous infusion is associated with sustained neuroendocrine activation and electrolyte imbalance that could potentially be reduced by intermittent administration.

The Diuretic Optimization Strategies Evaluation (DOSE) trial was a prospective, double-blind study in which researchers randomly assigned 308 subjects with ADHF to high-dose versus low-dose and continuous versus intermittent infusion of furosemide. This study did not reveal positive outcomes in either primary or secondary endpoints comparing continuous infusion to a bolus regimen. However, there were higher rates of acute kidney injury (AKI) in the high-dose arm [8]. Thus, equipoise remains on this issue. Our study aimed to evaluate the effects of continuous infusion of furosemide in comparison with a twice daily regimen at similar doses, on biomarker and clinical parameters.

## Methods

### Study design

**Figure 1:**
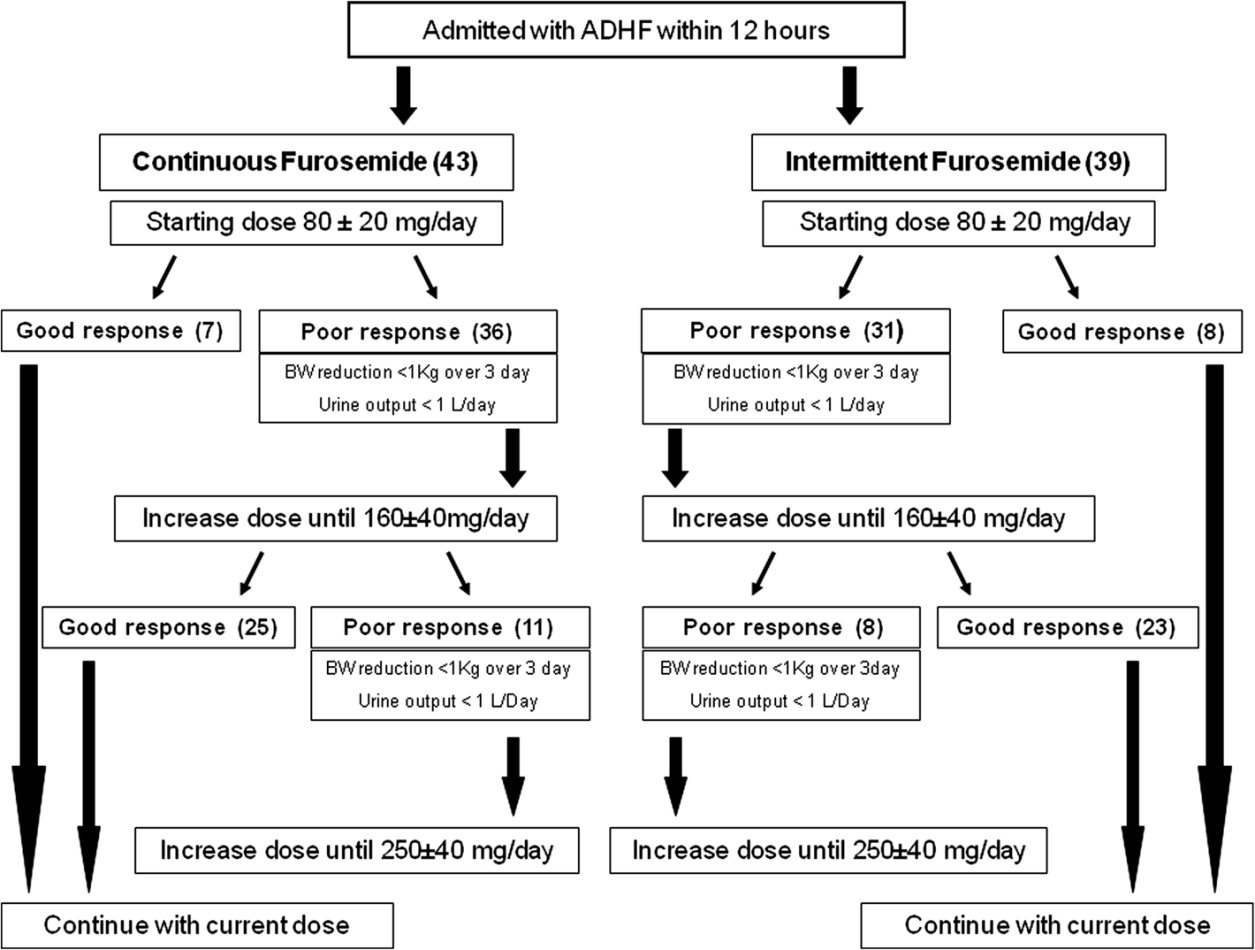
**Algorithm of diuretic treatment during randomization and study period: in both arms escalation doses were decided upon based on diuretic response, doubling previous dose administration in a step by step protocol.** ADHF, acute decompensated heart failure; BW, body weight.

This was a prospective, randomized, open label, double-blind study comparing continuous with intermittent infusion of furosemide in patients admitted with a diagnosis of ADHF into our tertiary-care medical center. The current pilot study design was planned to anticipate a larger multicenter trial able to definitively evaluate most optimal loop diuretic strategies in patients with ADHF. Patients were enrolled consecutively from the Department of Internal Medicine, Cardiology Section Center (Siena, Italy) from April 2010 to November 2012. All patients enrolled were submitted to continuous electrocardiography and blood pressure monitoring as well as regular measurements of urine output. We hypothesized that continuous infusion treatment could be superior to bolus intermittent administration to relieve congestion, increase diuresis and decrease B-type natriuretic peptide (BNP) levels.Patients were eligible if they were admitted with a primary diagnosis of ADHF, could be randomized within 12 h after hospital presentation and had evidence of volume overload (pulmonary congestion on chest radiography or significant increase in BNP levels). Some patients received noninvasive ventilation support before randomization (10 in the continuous and 7 in the bolus group). The cumulative daily dose of intravenous furosemide to be given in the initial 12 h was decided upon by the attending physician. Patients were then randomized in a 1:1 ratio using a computer-generated scheme into either twice-daily bolus injections or continuous infusion (mixed as a 1:1 ratio in 5% dextrose in water), for a time period ranging from 72 to 120 h. Boluses of furosemide were administered in 100 ml of water solution in one hour. The mean daily diuretic dosage was similar in both groups as a result of the protocol (Figure 1).

Before randomization, renal function parameters and BNP levels were measured in all patients. Therefore, all patients were submitted to echocardiograpy and chest radiography to assess pulmonary congestion. Chest radiography was also repeated promptly before treatment disruption to verify pulmonary congestion improvement. Subsequent titration of the furosemide dosage was guided by a dose-escalation algorithm based on the patient’s response to the treatment (body weight (BW) loss <1 kg from the starting enrollment to the third day or urine output <1,000 mL/day), signs of recovery (decreased pulmonary rales, venous jugular congestion, additive heart sounds, improvement in pulmonary congestion on chest radiography), and/or by important changes in renal function, such as a sudden increase of creatinine >0.8 mg/dl compared to the baseline value or hypokalemia below 3.2 mEq/L. The specific doses of furosemide and the use of additional agents to manage ADHF (dopamine, intravenous (IV) vasodilators, hypertonic saline infusions for hyponatremia) were decided upon based on the above cited laboratory and clinical parameters, with daily dosages adjusted during the infusion periods. Specifically in the case of poor initial diuretic response, the furosemide dosage was doubled. In case of diuretic resistance, defined as diuresis below 1,000 mL/day, the dosage was escalated up to 250 mg/day.

Supplementary treatment was left to the discretion of the treating physician. All patients took angiotensin-converting enzyme inhibitors or angiotensin receptor blockers and nitrates. No thiazide diuretics, nesiritide, or arginine vasopressin antagonists were administered during the hospitalization period. Hypertonic saline solution was administered to the patients who developed hyponatremia (serum Na values <128 mEq/L) during treatment with the goal of restoring plasma sodium values up to 134 mEq/L. For this reason Na concentration was monitored each day during the infusion period. The hypertonic saline solution consisted of 20 mEq of NaCl in 500 mL saline solutions (0.9% of NaCl). Infusion was administered at 80 cc/h once or twice a day, depending on the Na value [9]. Dopamine infusions were administered to patients with systolic blood pressure <90 mmHg with the goal of restoring systolic values up to 100 mmHg maintaining diuretic infusion. Inotrope therapy was stopped when blood pressure values were sustained at approximately 105 mmHg during 12 sequential hours for four consecutive measurements.

The frequency of laboratory tests to evaluate electrolyte balance and renal function during hospitalization was at the discretion of the attending physician but was guided by a dose-escalation algorithm. This trial was approved by our hospital’s Institutional Review Board of Siena and all patients gave their signed informed consent. This trial was registered and regularly updated in ClinicalTrials.gov with Identifier number: NCT01441245.

### Inclusion criteria

Patients over age 18 years of age were screened if they met diagnostic criteria for ADHF due to systolic dysfunction and left ventricular ejection fraction (LVEF) <45% by exhibiting at least one of the following symptoms at rest: dyspnea, orthopnea, peripheral edema or major fatigue and at least two clinical signs including rales, pulmonary congestion on chest radiography, jugular vein dilatation, or a third heart sound. Elevations in blood BNP >400 pg/mL were considered supportive for a diagnosis of ADHF.

### Exclusion criteria

Patients were excluded if they had received more than two IV doses of furosemide or any continuous infusion of furosemide one month before randomization, if they had end-stage renal disease or the need for renal replacement therapy (dialysis or ultrafiltration), isolated diastolic dysfunction with LVEF >45% or recent myocardial infarction within thirty days of screening. Patients with a systolic blood pressure <80 mm Hg or with serum creatinine levels >4.0 mg/dL were also excluded, as well as patients who received recent intravenous iodinated contrast.

### Laboratory analysis

Complete blood analysis with hemoglobin concentration, hematocrit, red blood cell count, serum creatinine, sodium, and potassium were performed at the time of admission to determine the baseline criteria, and subsequent testing was performed each day and again at the time of discharge. The estimated glomerular filtration rate (eGFR) was calculated using the four-variable formula, modification of diet in renal disease (MDRD). Mild kidney dysfunction was defined by eGFR less than 60 mL/min/1.73 m^2^, and moderate kidney dysfunction as eGFR 15 to 29 mL/min/1.73 m^2^ based on chronic kidney disease (CKD) stages. A rise in serum creatinine ≥0.3 mg/dl or urine output <0.5 mL/kg/h × 6 h was used according to conventional criteria to define AKI [10]. This measurement was calculated from admission to the end of diuretic infusion treatment. Plasma BNP was measured at baseline and at the end of infusion period using an immunofluorescence assay manufactured by Inverness (San Diego, CA, USA). The analytic sensitivity of the assay is <5 pg/mL and the upper limit of normal is considered to be <100 pg/mL.

### In-hospital outcomes

The co-primary endpoints at discharge were the mean paired changes in creatinine, eGFR, body weight (BW), and BNP. Additional in-hospital outcomes assessed included the rate of AKI, the use of hypertonic saline and inotropic agents, and length of hospital stay.

### Late outcomes

Death and rehospitalization because of worsening heart failure were captured up to 6 months after discharge. If the hospitalization was for heart-failure-related events such as pump failure, acute coronary syndromes complicated by heart failure, ventricular arrhythmias associated with left ventricular dysfunction, or heart failure associated with worsened renal function, for analytical purposes these were all considered as hospitalization for heart failure.

### Sample size estimation

The sample size used was preliminarily calculated on the basis of three co-primary endpoints including the following assumptions: 1) a 30% or greater effect size in the difference between mean paired changes in continuous co-primary endpoints (eGFR, creatinine, and BNP); SD data from each group no greater than 20%; 2) alpha = 0.05 two-tailed, 3) power (1-beta) = 80%. Thus, the calculated sample size was 52 subjects (26 in each group) and we assumed no patients would withdraw or be lost during the hospitalization. We also anticipated that 20% of subjects could be lost to follow up after hospital discharge.

### Statistical analysis

All data were analyzed with intention-to-treat principles. Continuous variables were expressed as mean ± SD and compared using the Student *t*-test (unpaired and paired as appropriate) for independent groups if normally distributed; normality was assessed by the Kolmogorov-Smirnov test. Analysis of variance was done by Levene’s test, and if it was breached Welch’s correction was used. Cox regression analysis was used to assess the independent relationship between the two methods of furosemide infusion for the outcome of rehospitalization or death, with adjustment for age, gender, creatinine, eGFR, and BNP at baseline, the use of hyperosmolar solution, dopamine infusion, and the development of AKI. Kaplan-Meier methods were employed to generate survival plots that were compared using a log-rank test for time to first hospitalization, death, or the composite. All statistical tests were two-tailed, with a *P*-value <0.05 considered significant. All the analyses were performed by using the SPSS 20.0 for Windows (SPSS Inc, Chicago, IL, USA).

## Results

### Baseline characteristics

A total of 128 patients were consecutively admitted and screened with diagnosis of ADHF; of these patients 22 were excluded for preserved ejection fraction, 8 for recent myocardial infarction, 8 for incomplete laboratory assessments at baseline, and 6 for severe renal disease. Of the 84 patients 2 were subsequently excluded because of missing data (weight, and BNP). Thus, 82 patients met the inclusion criteria and were randomly assigned to one of the two groups: continuous infusion, n = 43 or bolus therapy, n = 39 (Figure 1). The median time from presentation to randomization was 9 ± 3 h, and the median duration of study-drug administration was 112 ± 24 h. Table 1 provides patient’ characteristics in each group at the time of enrollment. The overall age was 72 ± 8 years, LVEF 35 ± 10%, serum creatinine 1.6 ± 0.5 mg/dl, and plasma BNP level 1156 ± 640 pg/Ml, with no significant differences between the randomized arms. The mean cumulative daily doses of furosemide chosen by the attending physicians were similar in both arms (continuous infusion: 170 ± 70 mg/day, bolus therapy 160 ± 80 mg/day).

**Table 1:** Baseline characteristics of the study population

	**Continuous infusion (n = 43)**	**Bolus (n = 39)**
Age (years)	80 ± 4	79 ± 5
Sex		
Female	24	18
Male	19	21
Baseline weight (kg)	72 ± 7	69.7 ± 10
Blood pressure (mmHg)	142/87	145/86
Heart rate (beats/minute)	102 ± 12	98 ± 16
Cardiac disease		
Coronary artery disease	24	21
Idiopathic cardiomyopathy	7	7
Hypertrophic cardiomiopathy	4	6
Valvular disease	8	5
Baseline creatinine (mg/dl)	1.62 ± 0.5	1.52 ± 0.4
BUN	100.60 ± 60	69.2 ± 31
eGFR (mL/min/1.73 m2))	43.2 ± 7.6	45.7 ± 8.7
Serum sodium (mEq/L)	137.2 ± 5	138 ± 5
Serum potassium (mEq/L)	4.19 ± 0.4	4.26 ± 0.5
Left ventricular ejection fraction (%)	34.3 ± 10	35.8 ± 8
LV internal diastolic diameter (mm)	68 ± 8	66 ± 9
LV internal systolic diameter (mm)	48 ± 10	45 ± 8
Estimated Pulmonary Artery (PA) systolic pressure (mmHg)	50 ± 6	48 ± 5
Signs of congestion		
Elevated jugular venous pressure	16	18
Additive heart sound	11	13
Peripheral edema	33	30
Pulmonary rales	38	35
Coronary risk factors (%)		
Diabetes mellitus	55.2	61.1
Hypertension	89.4	87.9
Dyslipidemia	72.4	75
Previous Coronary artery disease (CAD)	46.2	49.4
Atrial fibrillation (%)	36.6	41.3
Baseline BNP (pg/mL)	1204 ± 693	1099 ± 571
Previous therapy		
ACE-inhibitors	38	33
β-Blockers	22	21
Nitrates	25	26
Diuretics	39	35
Angiotensin receptor blockers	5	7
Digoxin	13	11
Aldosterone antagonist	15	12

Results are presented as mean ± SD or number, unless stated otherwise. BUN, blood urea nitrogen; eGFR, estimated glomerular filtration rate; LV, Left Ventricular; BNP, B-type natriuretic peptide.

### Laboratory values and urine output

At the end of treatment period, continuous infusions resulted in greater urine output (2295 ± 755 versus 2090 ± 421 mL, *P* <0.002); higher achieved serum creatinine values (1.78 ± 0.6 versus 1.34 ± 0.3 mg/dl *P* <0.0001), lower eGFR (40.6 ± 10.5 versus 50.4 ± 11.4 mL/min/1.73 m^2^, *P* <0.01), and higher blood urea nitrogen levels (100 ± 60 vs 69 ± 31 mg/dl, *P* <0.02). After the randomized treatment period of approximately 120 hours, the mean plasma BNP was lower in the continuous infusion arm (723 ± 497 versus 822 ± 548 pg/Ml, *P* = 0.05). By hospital discharge, there were lower potassium levels in the continuous arm (3.6 ± 0.8 versus 4.0 ± 0.7 mEq/L, *P* <0.04), reductions from baseline (-0.5 ± 1.4 versus -0.3 ± 0.9 mEq/L), however there were no significant differences in serum sodium (+1 ± 6 versus -3 ± 7 mEq/L) (Table 2).

**Table 2:** Comparison of biochemical measures and urine output after the randomized treatment period of approximately 120 h

	**Continuous infusion**	**Bolus**	***P*-value**
Urine output/24 h (mL)	2295 ± 775	2090 ± 421	<0.002
Serum creatinine (mg/dl)	1.78 ± 0.6	1.34 ± 0.3	<0.0001
eGFR (mL/min/1.73 m^2^)	40.6 ± 10.5	50.4 ± 11.4	<0.01
BUN (mg/dl)	100 ± 60	69 ± 31	<0.02
BNP (pg/mL)	723 ± 497	822 ± 548	<0.05
Serum sodium (mEq/L)	138 ± 4	135 ± 16	NS
Serum potassium (mEq/L)	3.6 ± 0.8	4.0 ± 0.7	<0.04

Results are presented as mean ± SD**.** eGFR, estimated glomerular filtration rate; BUN, blood urea nitrogen; BNP, B-type natriuretic peptide; NS, not significant.

### Primary endpoints

The mean change in serum creatinine was higher (+0.8 ± 0.4 versus -0.8 ± 0.3 mg/dl, *P* <0.01), and eGFR lower (-9 ± 7 versus +5 ± 6 mL/min/1.73 m^2^, *P* <0.05) in the continuous infusion arm. However, the mean reduction in BNP concentration from baseline to discharge was significantly greater with the continuous infusion compared to the bolus infusion (-576 ± 655 versus -181 ± 527 pg/mL, *P* = 0.02) (Table 3).

**Table 3:** Co-primary endpoints expressed as change from baseline to discharge in values

	**Confinuous infusion**	**Bolus**	***P*-value**
Δ Serum creatinine (mg/dl)	+0.8 ± 0,4	-0.8 ± 0.3	<0.01
Δ eGFR (mL/min/173 m^2^)	-9 ± 7	+5 ± 6	<0.05
Δ BNP (pg/mL)	-576 ± 655	-181 ± 527	0.02

Results are presented as mean ± SD. Δ: mean change from admission to discharge, Difference; eGFR, estimated glomerular filtration rate; BNP, B-type natriuretic peptide.

**Figure 2:**
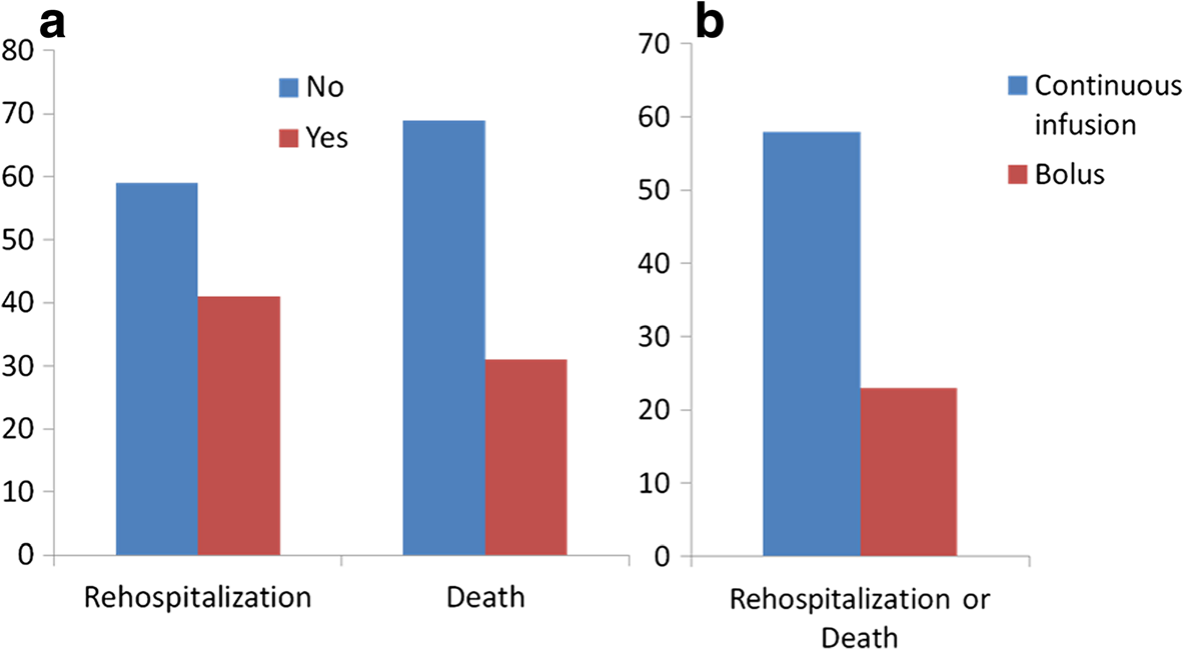
Percentage of rehospitalization and death in all population (a); comparison of adverse events between continuous and bolus groups during 6-months follow-up period (b).

As for the secondary outcomes, the incidence of AKI was similar (continuous arm 10 patients (22%) and bolus arm 6 patients (15%) *P* = 0.3) for the two groups. There was more frequent use of hypertonic saline solutions for hyponatremia (33% versus 18%, *P* <0.01), intravenous dopamine infusions (35% versus 23%, *P* = 0.02), and the hospital length of stay was longer in the continuous infusion group (14.3 ± 5 versus 11.5 ± 4 days, *P* <0.03) (Table 4). There was no significant difference in the degree of weight loss -4,1 ± 1,9 versus -3,5 ± 2,4 kg, p = 0.23. A total of 26 patients died (31%) and 35 (41%) had a new hospitalization during follow up. At 6 months there were higher rates of re-admission or death in the continuous infusion group, with 58% versus 23%, *P* = 0.001 (Figure 2).

**Table 4:** Secondary endpoints in the continuous infusion versus bolus arm

	**Continuous infusion**	**Bolus**	***P*-value**
Acute kidney injury	22%	15%	0.30
Hypertonic saline solution	33%	18%	0.01
Inotropes infusion	35%	23%	0.02
Length of hospital stay (days), mean ± SD	14 ± 5	11 ± 5	<0.03
Death or rehospitalization	58%	23%	0.001
Weight loss (kg), mean ± SD	-4.1 ± 1,9	-3.5 ± 2.4	0.23

### Multivariate results and late outcome

**Figure 3:**
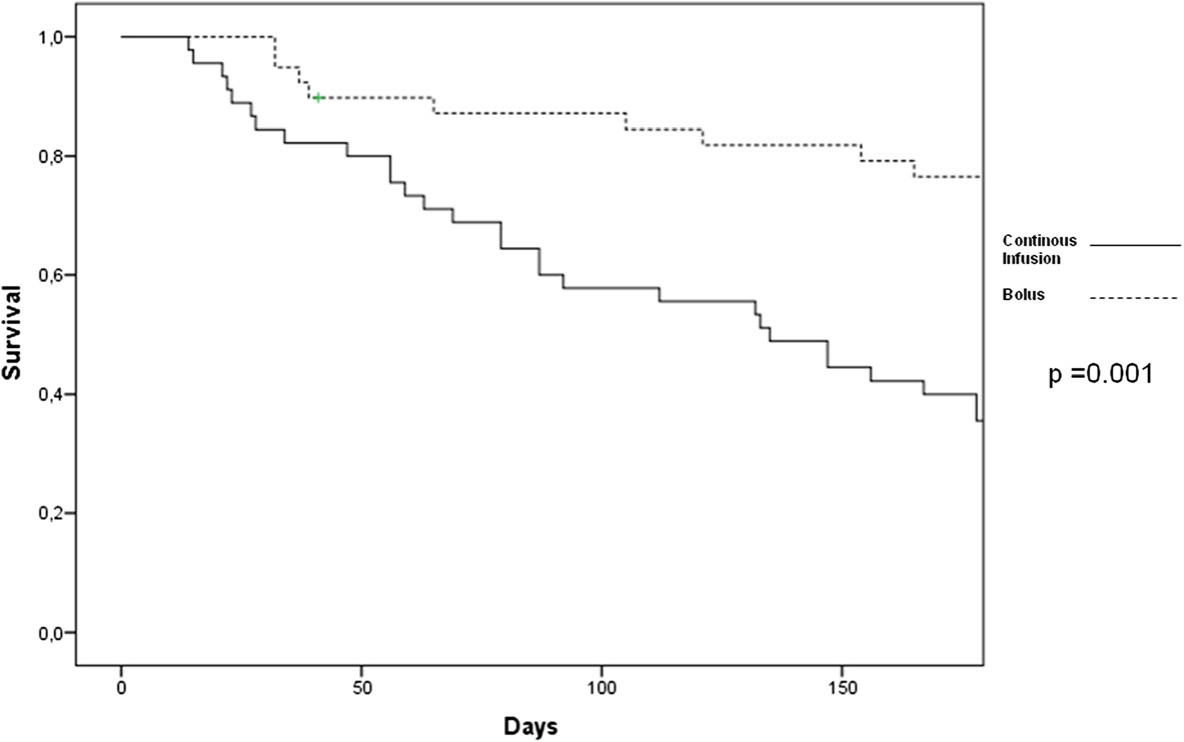
Kaplan Meier curves for the risk of rehospitalization or death at 180 days in those randomized to continuous (solid line) and bolus loop diuretics (broken line).

Univariate analysis for the composite outcome of rehospitalization or death at 6 months found that blood urea nitrogen (BUN) and randomization to continuous loop diuretics were the only baseline variables that were significantly associated with poor outcome. Additionally, serum creatinine, eGFR, and plasma BNP after the randomized treatment period (approximately 120 h) were associated with rehospitalization or death as shown in Table 5. When these variables were tested in a Cox proportional hazards model which included age, gender, baseline creatinine, eGFR, BUN and BNP, use of hyperosmolar solutions, dopamine infusions, eGFR after the treatment period, and the development of AKI, only randomization to continuous infusion (hazard ratio (HR) = 2.57, 95% CI 1.01, 6.58, *P* = 0.04), and after the treatment period (approximately 120 h) the serum creatinine above 1.5 mg/dl, (HR = 6.40, 95% CI 1.25, 32.62, *P* = 0.02), and BNP above 500 pg/mL, (HR = 1.01, 95% CI 1.00, 1.02, *P* = 0.04), remained significantly associated with rehospitalization or death.The Kaplan-Meier curve showed that in continuous arm there was an increased events rate during the 180-day observational period after discharge (Figure 3).

**Table 5:** Univariate and multivariate hazard ratios (HR) for rehospitalization or death at six months

**Rehospitalization or Death**				
	**Univariate**		**Multivariate**	
**Variable**	**HR (95** ***% *** **CI of HR)**	***P*-value**	**HR**^**a**^**(95** ***% *** **CI of HR)**	***P*-value**
BUN	1.01 (1.00, 1.02)	0.03	1.00 (0.99, 1.01)	NS
BNP AT*	1.01 (1.00, 1.02)	0.03	1.01 (1.00, 1.02)	0.04
eGFR AT*	0.98 (0.94, 1.03)	NS	1.06 (0.97, 1.15)	NS
Creatinine AT*	2.43 (0.94, 6.35)	NS	6.40 (1.25, 32.62)	0.02
Continuous vs bolus	2.91 (1.28, 6.63)	0.01	2.57 (1.01, 6.58)	0.04

*After treatment. ^**a**^Multivariate analysis adjusted for age, gender, baseline creatinine, eGFR BUN and BNP, use of hyperosmolar solutions, dopamine infusions, eGFR AT, the development of acute kidney injury. eGFR, estimated glomerular filtration rate; BUN, blood urea nitrogen; BNP, B-type natriuretic peptide; NS, not significant.

## Discussion

Loop diuretic therapy is considered the cornerstone for heart failure (HF) management, particularly during episodes of acute decompensation. More than 90% of patients admitted for HF are treated with this drug [2,3]. Although mentioned in guidelines for ADHF, there is little evidence to support a preferred dose, route of administration, or method of intravenous infusion [6,11]. Furthermore, observational studies have demonstrated that higher cumulative doses of loop diuretics have been associated with worsened outcomes [12,13]. Thus, our trial had an excellent setting to allow clinicians to choose a cumulative daily dose of diuretics and then randomize patients to a continuous versus bolus administration of loop diuretics.

### Continuous versus intermittent loop diuretic administration

We found a few clinical advantages to the use of continuously infused loop diuretics in that there appeared to be a greater urine output after 24 h and a greater reduction in BNP after approximately 5 days. All other clinical factors favored the use of intermittent twice daily bolus administration including the change in serum creatinine, eGFR, need for the use of hypertonic saline for hyponatremia, use of inotropic agents, length of hospital stay, and rehospitalization or death at 6 months.

Importantly, after we adjusted for intermediate outcomes and physician responses to clinical changes, we found that randomization to continuous infusion therapy was strongly associated with rehospitalization and death at 6 months. The power of this evaluation is low due to low numbers of subjects Thus, despite any theoretical advantages of continuous administration, we found no clinical benefit; in fact there was evidence of harm with this approach. All of these effects related either to greater diuresis or preexisting undocumented morbidity would be expected to worsen outcomes. Our findings revealed a bimodal effect of continuous infusion: despite a significant decrease in BNP levels we found an increase of eGFR and creatinine, such a trend seems to reflect the late outcome. The current course should evidence the prognostic impact of AKI compared to BNP reduction during hospitalization could have more unfavorable impact in our population. Findings of multivariate analysis appear in line with the current observation: both BNP and creatinine at discharge revealed a prognostic impact, although creatinine together with type of treatment, provide evidence of a more significant value. Moreover, patients in the continuous infusion arm were more often submitted to dobutamine therapy, and this could have biased the outcome findings.

### Pharmacology of loop diuretics

In patients with ADHF treated with loop diuretics, compensatory pathophysiologic mechanisms to maintain vascular resistance, such as nonosmotic stimulation of vasopressin secretion and activation of the renin-angiotensin system, have been observed. Because intravenous loop diuretics, as observed in our study, result in an initial high-volume diuresis, the plasma refill of fluid from the extravascular space may be exceeded by early volume depletion, and thus create a risk for AKI [14]. Additionally, loop diuretics may directly impair renal function by reducing renal blood flow resulting in redistribution within the kidney and inducing a reduction in the effective filtration fraction [7,15]. More recent analyses, however, suggest that aggressive use of diuretics may be necessary in severe cases and that adverse effects may result from disease severity [16,17]. Thus, we recognize that in ADHF, higher doses of intravenous loop diuretics may be unavoidable, therefore, the remaining issue is how best to administer the therapy. In theory, continuous administration could be associated with higher drug concentrations at the loop of Henle, reducing energy requirements of the cells at the medullary level and consequently providing protection during hypoxic states. All these mechanisms lead to a resting state and a decrease in tubuloglomerular feedback. Therefore, a continuous modality should provide more constant urine output, less variation of intravascular volume, and less sodium reabsorption. These beneficial effects must be weighed against more sustained neuroendocrine activation, greater counter-regulatory attempts to increase sodium and water reabsorption, and efferent arteriole vasoconstriction for a prolonged period of time [15,17]. A recent Cochrane analysis was consistent with this rationale by demonstrating that continuous infusions ultimately resulted in lower urine outputs and greater rates of adverse events; unfortunately, no data were reported on long-term mortality or post-discharge events [18].

### Reduction in renal filtration, B-type natriuretic peptide, and outcomes

We found that larger volumes of diuresis were associated with greater elevations in serum creatinine suggesting that the rate of salt and water loss exceeded that of plasma refill from the extra vascular compartment. A very similar observation was made in the Evaluation Study of Congestive Heart Failure and Pulmonary Artery Catheterization Effectiveness (ESCAPE) trial [19]. We found that despite greater reductions in BNP, there was worsened renal filtration function and no change in body weight. Studies have demonstrated that reductions in BNP are in general associated with improved short- and longer-term clinical outcomes, particularly when the reduction is more than 30% respect to admission level [20-22]. We found the differential reduction in BNP between the two groups was modest, and there was a wide range of BNP levels both before and after treatment. We observed a bimodal laboratory trend in the two arms: the continuous arm showed greater diuresis with better BNP reduction, Intermittent administration revealed less worsening renal function and less need for additive therapy. Thus, BNP is a single clinical parameter co-dependent on many variables related to left ventricular wall tension, degree of neurohormonal activation, and renal filtration function [23-25]. While many studies have focused on baseline values at the time of admission, we found that serum creatinine and BNP after the first 5 days of hospitalization was more significantly associated with 6-month outcomes [26-28]. Thus, our data suggest that in a clinical trial setting, the reduction in BNP does not appear to be a surrogate for improved renal or clinical outcomes in patients with ADHF, whereas basing of our finding, worsening renal function during hospitalization as well as basal renal dysfunction are two parameters that deserve better attention in clinical practice.

### Previous management studies

The DOSE trial provided important information in determining the best method of administration of diuretics in this setting. Our results are partly in accordance with the DOSE trial which did not find superiority in either the dose (high versus low) or administration (infusion versus bolus) arms of a two-by-two factorial study [8]. Like DOSE, our trial found that continuous infusions at higher doses tended to result in a greater incidence of AKI. Our trial differed from DOSE in that our patients had greater degrees of baseline renal impairment, the loop diuretics were given for longer durations under the study protocol, and importantly, we found that the continuous infusion was associated with the need for more in-hospital therapies (inotropic infusions for hypotension and hypertonic saline for hyponatremia). This probably justifies a longer in-hospital stay together with worst basal conditions and older age; in respect to the DOSE trial patients moreover, most of them had comorbidities (as renal insufficiency anemic status and pulmonary disease). In addition, we found higher rates of rehospitalization and death in the continuous infusion arm. We did not evaluate symptom relief as in the DOSE trial, thus, there may have been a short-term differential strategy that we missed with our design. We preferred to use laboratory parameters and the endpoint of urine output because the symptom-stair is often misleading and does not perfectly reflect the congestion status and effective clinical improvement. Therefore the endpoints considered were demonstrated to have an effective impact on early outcome. Finally they may permit better tailoring of the dosing regimen for each patient during protocol escalation, avoiding the side effects of therapy. Other smaller studies comparing either dose or administration therapy have not yielded important conclusions beyond what can be reached from our trial or the DOSE trial, thus, additional research on optimal, inpatient treatment strategies is warranted in ADHF as well as in acute cardiorenal syndrome [18,29-32].

### Limitations

Our trial has all the limitations of small, single-center randomized, pilot trials; however, except for the DOSE HF trial it is the largest study comparing the modality of loop diuretic administration in the acute HF setting. As a practical consideration in management, we had the attending physicians choose the cumulative daily dose of loop diuretic based on their judgment, and the randomization dictated the method of infusion, thus, treatment bias on dose as well as dose adjustments was not completely handled in the protocol. Furthermore, we did not use a double-dummy design to blind the method of infusion. We did not have statistical power to evaluate differences in clinical outcomes during the hospitalization or at 6 months follow up. The multivariate analysis, including several risk factors and comorbidities, should be interpreted with caution because of the relativey small sample size. As in most randomized trials of ADHF, we did not change the background therapy in the protocol, thus, there was inherent non-uniform standard therapy (that is, nitrate, ACE-inhibitors, beta-blockers). As one of the outcomes, renal filtration function was calculated by the MDRD formula, which could have been influenced by several variables; the gold-standard calculation would be the direct measurement of creatinine clearance, which was not done in our study. Our findings arise from a pilot study that needs to be confirmed in large sample size, and for this reason we are pursuing the enrollment, and we intend to enlarge our protocol for a multicenter trial. We did not measure novel biomarkers, assess urinary sodium, or perform other assays that would have helped us in understanding why continuous infusions led to greater reductions in eGFR and worsened outcomes. Finally, our findings cannot be extended to patients with newly diagnosed HF or to those with lower diuretic requirements or perhaps better baseline renal function, where the response to diuretic strategies may be different.

## Conclusions

Continuous infusions of loop diuretics in patients with ADHF appear to provide more efficient diuresis, together with better reduction of BNP levels in comparison with bolus therapy during the in-hospital period. However, this approach is associated with greater reductions in eGFR, needing of additional therapy for hypotension and hyponatremia, and longer hospitalization. These events appear to translate into higher rates of rehospitalization and death at 180 days. A larger multicenter study utilizing a more sophisticated assessment of volume overload and congestion, measurement of plasma refill from the extravascular compartment, and novel markers of both renal function and damage is needed to further understand how loop diuretics affect impact patients with ADHF and their short- and longer-term clinical outcomes. It is possible that degrees of physiologic tailoring of diuretic dose and administration are the next testable hypotheses in randomized trials that aim to improve outcomes in this population.

## Key messages

• Continuous infusion of loop diuretics is associated with greater urine output and greater reduction in BNP in respect to intermittent infusion during hospitalization in patients with ADHF

• In this population continuous infusion of loop diuretics is associated with an increased rate of AKI before discharge

• An increased use of additional therapy has been observed in the continuous infusion arm because of increased rates of hypotension and hyponatremia

• An increased rate of worsened renal function in the continuous arm appears related to impaired long-term outcome in patients with ADHF

• A larger multicenter study utilizing a more tailored diuretic dose and administration could clarify this bimodal trend

## Abbreviations

ADHF: acute decompesated heart failure; AKI: acute kidney injury; BNP: B-type natriuretic peptide; BUN: blood urea nitrogen; BW: body weight; CKD: chronic kidney disease; eGFR: estimated glomerular filtration rate; HF: heart failure; HR: hazard ratio; IV: intravenous; LVEF: left ventricular ejection fraction; MDRD: modification of diet in renal disease.

## Competing interests

The authors declare that they have no competing interests.

## Authors’ contributions

AP participated in the conception, design, drafting of the work and final approval of the manuscript version to be published. MP participated in the acquisition of data, analysis, drafting of the work and final approval of the manuscript version to be published. GR participated in the statistical analysis, interpretation of data, drafting of the work and final approval of the manuscript version to be published. GM participated in the acquisition of data, analysis, drafting of the work and final approval of the manuscript version to be published. BF participated in the analysis of the blood samples, acquisition of data, drafting of the work and final approval of the manuscript version to be published. MSC participated in the analysis of the blood samples, acquisition of data, drafting of the work and final approval of the manuscript version to be published. MG participated in the language revision, acquisition of data, drafting of the work and final approval of the manuscript version to be published. RN participated in the conception, financial support, drafting of the work and final approval of the manuscript version to be published. PAM participated in the conception, interpretation of data for the work, manuscript writing, critical revision of the work and final approval of the manuscript version to be published. CR participated in the conception, drafting of the manuscript or revising it critically for important intellectual content and final approval of the manuscript version to be published. All authors read and approved the final manuscript version.
